# Insights Into Extracellular Vesicles as Biomarker of NAFLD Pathogenesis

**DOI:** 10.3389/fmed.2020.00395

**Published:** 2020-08-11

**Authors:** Irma Garcia-Martinez, Rosa Alen, Patricia Rada, Angela M. Valverde

**Affiliations:** ^1^Instituto de Investigaciones Biomédicas Alberto Sols (CSIC/UAM), Madrid, Spain; ^2^Centro de Investigación Biomédica en Red de Diabetes y Enfermedades Metabólicas Asociadas (CIBERdem, ISCIII), Madrid, Spain

**Keywords:** nonalcoholic fatty liver disease, hepatocyte, lipotoxicity, inflammation, intercellular communication, extracellular vesicles, biomarkers

## Abstract

Nonalcoholic fatty liver disease (NAFLD) is the most common cause of chronic liver disease around the world estimated to affect up to one-third of the adult population and is expected to continue rising in the coming years. Nonalcoholic fatty liver disease is considered as the hepatic manifestation of the metabolic syndrome because it is strongly associated with obesity, insulin resistance, type 2 diabetes mellitus, and cardiovascular complications. Despite its high prevalence, factors leading to NAFLD progression from simple steatosis to nonalcoholic steatohepatitis, cirrhosis, and, ultimately hepatocellular carcinoma remain poorly understood. To date, no treatment has proven efficacy, and also no reliable method is currently available for diagnosis or staging of NAFLD beyond the highly invasive liver biopsy. Recently, extracellular vesicles (EVs) have emerged as potential candidate biomarkers for the diagnosis of NAFLD. Extracellular vesicles are circulating, cell-derived vesicles containing proteins and nucleic acids, among other components, that interact with and trigger a plethora of responses in neighbor or distant target cells. Several mechanisms implicated in NAFLD progression, such as inflammation, fibrosis, and angiogenesis, all related to metabolic syndrome–associated lipotoxicity, trigger EV production and release by liver cells. As hepatocytes represent ~80% of the liver volume, in this review we will focus on hepatocyte-derived EVs as drivers of the interactome between different liver cell types in NAFLD pathogenesis, as well as in their role as noninvasive biomarkers for NAFLD diagnosis and progression. Based on that, we will highlight the research that is currently available on EVs in this topic, the current limitations, and future directions for implementation in a clinical setting as biomarkers or targets of liver disease.

## Introduction

Nonalcoholic fatty liver disease (NAFLD) is the most common chronic liver disease worldwide, affecting 25% of the global adult population, especially in industrialized countries (1). In addition, NAFLD is also the most prevalent form of chronic liver disease in childhood, affecting ~10% of the general pediatric population (2, 3). The classic definition of NAFLD excludes excessive alcohol consumption, which is well known to cause alcoholic liver disease. Growing evidences support the hypothesis that NAFLD is the hepatic manifestation of metabolic syndrome, with insulin resistance as the common pathogenic factor (4). Although some genetic risk factors have been reported (5), the increase in body weight and the presence of several hallmarks of metabolic syndrome such as adiposity, hyperglycemia, dyslipidemia, and hypertension, may be key determinants in the pathogenesis of NAFLD.

Nonalcoholic fatty liver disease includes a wide signature of liver damage, extending from nonalcoholic fatty liver (NAFL) or steatosis toward nonalcoholic steatohepatitis (NASH), liver fibrosis, cirrhosis, and hepatocellular carcinoma (HCC), lately causing chronic hepatic insufficiency and the need for liver transplantation. Whereas, NAFL is defined by simple liver steatosis, NASH is characterized by the joint presence of steatosis and lobular inflammation with hepatocyte ballooning degeneration, with or without any fibrosis (6, 7). The underlying triggers and mechanisms for the development and progression of NAFLD, an issue under current investigation, are complex and multifactorial. Originally, the “two-hit hypothesis” was formulated in order to explain the progression from simple steatosis to NASH. According to this traditional point of view, the intrahepatic accumulation of lipids secondary to sedentary lifestyle, hypercaloric diets, obesity, and insulin resistance acts as the “first hit” sensitizing the hepatocytes to further injuries or insults. Proinflammatory cytokines, adipokines, bacterial endotoxins, mitochondrial dysfunction, oxidative stress, and/or endoplasmic reticulum (ER) stress represent the “second hit” for the progression to NASH. Subsequently, the “second hit” leads to hepatocyte injury, inflammation, and fibrosis. To date, the most widely accepted hypothesis is the “multiple-hit model” (8), because many other additional elements such as hormones/adipokines secreted from the adipose tissue, nutritional factors, gut microbiota, and genetic and epigenetic factors also contribute to the progression of this disease (9–13). However, liver fat accumulation, caused by obesity and insulin resistance, still seems to represent the first hit. In the adipose tissue, insulin resistance leads to an impaired suppression of lipolysis, causing triglyceride (TG) breakdown and a massive accumulation of free fatty acids (FFAs) and glycerol. Circulating FFAs are taken up by the hepatocytes and esterified into TGs. However, an excessive uptake of saturated FFAs may overwhelm the cellular capacity to store and esterify them into TG, leading to organelle dysfunction, cell injury (lipotoxicity), and apoptotic cell death (lipoapoptosis) of the hepatocytes, processes strongly associated with the progression from NAFLD to NASH.

In most cases, NASH typically develops asymptomatic until the disease progresses to end-stage liver disease at which liver transplantation is the only available therapeutic option. Therefore, early detection of NAFLD may be useful to identify those individuals with potentially silent progressive fatty liver disease. In some cases, the presence of NAFLD has been strongly suspected in individuals showing unexplained elevation of liver enzymes levels or evidence of steatosis by imaging. To date, liver biopsy remains the gold standard method for NAFLD diagnosis that classifies the state of the disease by histologic assessment. However, this highly invasive and harmful procedure to the patient cannot predict disease progression and often leads to late diagnosis.

At present, no pharmacological agents for the treatment of NASH have been currently approved by the US Food and Drug Administration. Therefore, lifestyle and dietary changes leading to weight loss are the first-line strategy to overcome NAFLD. Unfortunately, it has been demonstrated that a high number of patients regain most of the weight after a successful weight loss period, an effect likely due to the unavailability of a full multidisciplinary program focused on long-term weight maintenance for the patient (14, 15). For this reason, there is an urgent need to identify reliable noninvasive biomarkers specific for NAFLD and NASH diagnosis at an earlier time point at which lifestyle interventions and potential newly developed drugs can be used successfully.

To date, it is assumed that the lack of highly effective treatments may be due to the heterogeneity of the population with NAFLD with respect to its primary drivers and coexisting disease modifiers such as cardiovascular diseases (CVDs) or type 2 diabetes mellitus (T2DM). In fact, growing evidences suggest that, during NAFLD, a dichotomous classification of patients with or without NASH may not represent the full spectrum of disease progression due to the aforementioned modifying factors. Recently, the updated term MAFLD, metabolic dysfunction–associated fatty liver disease, has emerged to better define NAFLD pathogenesis and denotes the hepatic manifestation of a multisystem disorder that is heterogeneous in its underlying origin, progression, and outcomes (16, 17). MAFLD represents the overarching umbrella comprising multiple subtypes, but reflecting the dominant driver. As proposed, the criteria for diagnosis are based on evidence of hepatic steatosis in addition to one of the following three criteria, namely, overweight/obesity, presence of T2DM, or evidence of metabolic dysregulation, regardless of the amount of alcohol consumed. Therefore, the identification of the predominant drivers in each patient might allow the implementation of a personalized treatment to ensure the best response with low adverse side effects.

Recent advances in basic and translational research have provided insights into the pathogenic mechanisms driving the progression of NAFLD that involve parenchymal and nonparenchymal liver cells (18). Stressed or dying hepatocytes during lipotoxicity release intracellular molecules named damage-associated molecular patterns or DAMPs, which activate various cell types such as Kupffer cells (KCs), liver resident macrophages, neutrophils, and hepatic stellate cells (HSCs), boosting inflammation and fibrosis. It is noteworthy to highlight that the location of HSCs and KCs within the space of Disse facilitates their direct contact with other cell types including hepatocytes and liver sinusoidal endothelial cells (LSECs), thus promoting the intercellular transport of soluble mediators and cytokines. According to this, the progression from NASH to more advanced stages is the result of a complex intrahepatic interactome between different cell types via secreted factors, illustrating the complexity of cell–cell signaling in liver physiology and disease. Given this scenario, there is no a single therapeutic target for NAFLD treatment, which explains the lack of an effective therapy for the disease.

## Emerging Role of Extracellular Vesicles in Cell-to-Cell Communication

Through the years, intercellular communication has been thought to be mediated only by direct cell-to-cell interaction or secretion of soluble factors. Nonetheless, it is now well recognized that cells are also capable of releasing, in an evolutionally conserved manner, various types of membrane vesicles as a third type of cellular interactome. These vesicles are generally known as extracellular vesicles or “EVs” (19).

**Figure 1:**
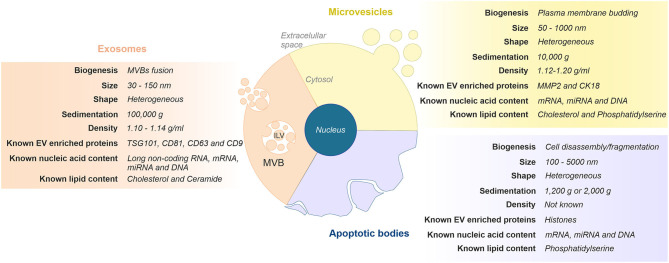
Classification and representative characteristics of extracellular vesicles (EVs) subtypes. Extracellular vesicles can be classified in three main subgroups based on their size and cellular biogenesis: exosomes, microvesicles, and apoptotic bodies. Exosomes are formed via the endosomal pathway and are released upon fusion of MVBs with the plasma membrane. Microvesicles are generated by the outward budding and fission of the plasma membrane. Apoptotic vesicles are released upon cell fragmentation during apoptotic cell death. ILV, intraluminal vesicle; MVB, multivesicular body.

The generic term “EVs” comprises a heterogeneous population of cell-released, nanometer-sized vesicles enclosed by a lipid bilayer membrane. Currently, EVs can be broadly classified into three main categories based on their size and cellular biogenesis: exosomes, microvesicles, and apoptotic bodies (19, 20). Briefly, exosomes are the smallest vesicles (30–150 nm) and are formed as intraluminal vesicles within multivesicular bodies (MVBs), which are released upon fusion with the plasma membrane. Microvesicles (50–1,000 nm) and apoptotic bodies (100–5,000 nm) are larger and formed by outward budding and fission of the plasma membrane, or when plasma membrane blebbing occurs during apoptosis, respectively. Figure 1 depicts the features commonly used to differentiate EVs subtypes.

Biogenesis of exosomes and microvesicles occurs at distinct sites within the cell. Also, they display similar morphology and overlapping size and share intracellular machinery in their formation. Therefore, this similar composition makes it difficult to identify. This, along with the lack of standardization of both isolation procedures and methods for their characterization, challenges current attempts to devise a more precise nomenclature (20, 21). In this regard, as the number of scientific publications in the field has increased in the last decade (22), the International Society of Extracellular Vesicles (ISEV: www.isev.org) published in 2014 the Minimal Information for Studies of Extracellular Vesicles (“MISEV”) guidelines (23) with the aim to unify the nomenclature and the methodologies for EVs studies worldwide. MISEV has been updated in 2018 (24).

Even though EVs are known since late 1980s (25, 26), they have recently remerged in the scientific community on biomedical research. Nowadays, the main interest in the EVs field is focused on their capacity to shuttle between cells, as well as in their specific bioactive cargo molecules such as nucleic acids (i.e., DNAs, RNAs, and noncoding RNAs such as miRNAs), proteins, lipids, sugars, and other metabolites that are transported in response to different stimuli. Of note, it is known that the process of cargo sorting is highly regulated; however, the mechanisms involved remain largely unknown (27). Therefore, each cell type can regulate EV production both quantitatively and qualitatively, depending on its physiological or pathological state. Furthermore, the same cell type may secrete heterogeneous populations of EVs if several mechanisms with distinct activators are involved (19, 20, 28).

Once released into the extracellular milieu, EVs can interact with nearby cells (cell-to-cell communication) or diffuse into bloodstream or other body fluids to act in distant organs (interorgan communication). Ultimately, EVs are able to transmit a unique package of information from donor-to-recipient cells, thereby eliciting functional responses and promoting phenotypic changes that will affect their physiological or pathological status (19, 20).

Extracellular vesicles need to selectively dock at the plasma membrane of specific target cells for triggering their phenotypic effects. In fact, all EVs bear surface molecules that allow them to be recognized by recipient cells. Several studies indicate that surface glycosylation patterns and exposed receptors and ligands (i.e., integrins) may be of relevance for EVs binding to target cells and, therefore, for their subsequent biodistribution (29, 30). Once attached to a target cell, EVs can initiate intracellular signaling pathways through the simple interaction with surface receptors or ligands. For example, EVs bearing surface ligands such as FasL, perforin, or tumor necrosis factor–related apoptosis-inducing ligand (TRAIL) are fully functional in inducing death receptor–mediated apoptosis (31–33). Nonetheless, EVs cargo delivery by vesicle internalization (endocytosis) or fusion with target cells is commonly needed for specific cellular responses. In this regard, EVs can release inside the recipient cell proteins, bioactive lipids, or even active signaling molecules, including enzymes, which activate a plethora of downstream signaling pathways. Moreover, EVs can also load active mRNAs and miRNAs that regulate gene expression through *de novo* translation or posttranslational regulation of target mRNAs, respectively (20, 34).

Extracellular vesicle–mediated intercellular communication is necessary to maintain cellular homeostasis and physiological functions, whereas alterations in this process could be an indicator of pathological states. The fact that EVs cargo can be modified under pathological conditions raises the question whether EVs might have a different biological role in health or disease. Hence, EVs could serve as potential therapeutic targets in the treatment of several pathologies. Moreover, because of disease-associated cargo and ubiquitous presence and stability of EVs in various human biofluids, they may also have clinical relevance as noninvasive biomarkers for disease detection and prognosis (35). On the other hand, EVs, either unmodified or engineered, have also generated considerable attention in the scientific community because of their potential use for therapeutic purposes (36). Extracellular vesicles are bioavailable, biocompatible, and resistant to RNases and proteases (37). These characteristics make them ideal vehicles for the delivery of drugs, proteins, miRNAs, silencing RNAs (siRNAs), and other molecules. Regarding to liver diseases as a major focus of this review, efforts have been focused in two major areas: on the one hand, the use of EVs as delivery vehicles of drugs to the liver (38) and, on the other, the use of EVs themselves as therapeutic agents to stimulate liver regeneration, modulate inflammation, reduce liver fibrosis, or block hepatocarcinogenesis (39, 40). However, several substantial challenges such as standardization of methodology and selection of the type of EVs for delivery still need to be solved before controlled clinical studies can be carried out (41, 42).

Identification and analysis of cell/tissue–specific molecular patterns is promising for diagnostic, prognostic, and therapeutic purposes. The tissue-specific protein composition of EVs provides opportunities to identify cell type–specific signatures to be used as diagnostic markers. Several protein screening methods available such as two-dimensional gel electrophoresis and mass spectrometry are time consuming, poorly suited for high-throughput screening of many samples, and their sensitivity and reproducibility may be limited. In this sense, Larssen et al. (43) showed that multiplex proximity extension assays (PEAs) are a powerful protein screening tool in EVs research. This technology allows identification, analysis, and validation of potential EVs-associated markers to identify with high specificity and sensitivity the protein profiles of EVs of different origins. Importantly, the ability of this technology to trace cellular origin could be extended to plasma-derived EVs, facilitating efficient and noninvasive diagnostic strategies at early stages of diseases. A limitation of the current study is the fact that the PEA panels are not available to all EV target cells. Thus, further investigation and optimization of PEA to be used in the screening of larger patient cohorts and additional body fluids are needed.

Accumulating evidence supports a role for EVs in a wide range of human diseases, including the spectrum of conditions associated with obesity and metabolic syndrome (44). Moreover, the abundance and the phenotype of blood-circulating EVs have been reported to change in obesity and associated disease states including insulin resistance, T2DM, and NAFLD (45). Several mechanisms implicated in NAFLD progression, such as inflammation, fibrosis, and angiogenesis, all related to metabolic syndrome–associated lipotoxicity, trigger EV production and release by the liver (45). On the one hand, EVs mediate local intercellular communication between the liver cells, thereby driving disease pathogenesis, and on the other, liver-derived EVs could affect distant tissues and organs upon their release to the bloodstream. Thus, liver-derived EVs have promise as biomarkers for diagnostic and prognostic purposes in patients (46). However, the identification of liver-derived EVs in circulation as indicative of metabolic alterations in this organ is still a challenge for basic and clinical researchers.

As mentioned above, NAFLD is not an isolated condition, and generally speaking, this disease occurs as a complication of other metabolic disorders. Therefore, multiple tissues may be affected, and consequently, the contribution of extrahepatic EVs during NAFLD cannot be excluded (i.e., adipocyte- or immune cells–derived EVs). Furthermore, most liver cell types produce EVs including hepatocytes, cholangiocytes, HSCs, and LSECs (35). Nonetheless, as 80% of the liver volume is composed by hepatocytes, their participation to the total pool of liver-derived EVs is likely the most relevant. Therefore, in this review, we will focus on hepatocyte-derived EVs (Hep-EVs) as drivers of NAFLD pathogenesis or noninvasive biomarkers for NAFLD diagnostic and prognostic.

## Role of Hepatocyte-Derived EVs in NAFLD

Lipotoxicity is one of the triggers of NAFLD progression because it is a process by which accumulation of toxic lipids species in hepatocytes such as saturated FFAs activates molecular pathways related to cellular stress that can result in cell death (47, 48). In this section, we will analyze different studies with evidences on lipotoxic hepatocyte injury that affects, or even drives, the responses of surrounding liver cells through the release of Hep-EVs. On the other hand, we will highlight the studies currently available that point to the participation of Hep-EVs in NAFLD-associated complications.

### Hep-EVs as Key Role in the Progression NAFLD to NASH

**Figure 2:**
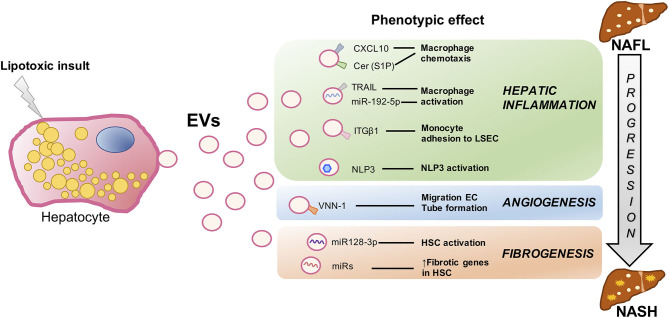
Signaling events mediated by extracellular vesicles during hepatocyte lipotoxicity. Hepatocyte injury induced by lipotoxicity triggers the release of EVs (Hep-EVs) that drive the response of surrounding cells playing an important role during NAFLD progression to NASH such as hepatic inflammation, dysregulated angiogenesis, and fibrosis. Recent *in vitro* and *in vivo* studies have defined multiple Hep-EV cargos responsible of different phenotypic effects in the target cells. CXCL10 and ceramide-enriched EVs mediate monocyte/macrophage chemotaxis to the liver, whereas TRAIL-enriched EVs and miR192-5p contribute to macrophage activation. ITGβ-enriched EVs regulate monocyte adhesion to LSECs, and Hep-EVs can also activate NLRP3 inflammasome. VNN1-1–bearing EVs mediate endothelial cell migration and tube formation and neovascularization, whereas miR-128-3p–laden EVs induce HSC proliferation and activation. EV, extracellular vesicles; CXCL10, C-X-C motif chemokine ligand 10; Cer, ceramide; S1P, sphingosine 1-phosphate; TRAIL, tumor necrosis factor–like apoptosis inducing ligand; ITGβ1, integrin β1, LSEC, liver sinusoidal endothelial cells; NLRP3, nucleotide-binding oligomerization domain-like receptor protein 3; VNN1, vanin-1; EC, endothelial cells; HSCs, hepatic stellate cells; NAFLD, nonalcoholic fatty liver; NASH, nonalcoholic steatohepatitis.

As we stated above, there are several key events closely interconnected involved in NAFLD progression to NASH such as inflammation, fibrosis, hepatocyte cell death, and dysregulated angiogenesis. All of these signs are related to metabolic syndrome–associated lipotoxicity which triggers EV production and release by the liver (45). Next, we will review in detail several studies which compile the role of proinflammatory, proangiogenic, and profibrotic Hep-EVs as pathogenic mediators during lipotoxicity in NAFLD (Figure 2).

#### Hep-EVs in Liver Inflammation

Recruitment of monocyte-derived macrophages into the liver contributes to the inflammatory response during NASH (49). However, how hepatocyte lipotoxicity promotes monocyte-derived macrophages chemotaxis, activation, and hepatic inflammation, all of these pathogenic processes being essential in the progression of NAFLD, also remains unclear.

Ibrahim et al. demonstrated that proapoptotic lipotoxic signaling triggered by mixed lineage kinase 3 (MLK3) induces the release of proinflammatory Hep-EVs enriched in potent C-X-C motif chemokine ligand 10 (CXCL10) that, in turn, lead to monocyte-derived macrophages chemotaxis to the liver and may activate KCs during NASH progression (50). Moreover, MLK3-deficient mice fed a fat-, fructose-, and cholesterol-enriched diet (FFC diet) were protected against development of dietary steatohepatitis. This beneficial effect was associated with a reduction in the number of total plasma EVs and EVs containing CXCL10 compared to the wild-type mice. In another study, Kakazu et al. illustrated that palmitate-induced Hep-EVs are enriched in C16:0 ceramide, a bioactive lipid specie generated from palmitate (51). C16:0 ceramide-enriched Hep-EVs were released from damaged hepatocytes in response to lipotoxicity, an effect mediated by the ER stress sensor inositol requiring enzyme 1α (IRE1α). Palmitate-induced EVs were chemoattractive toward macrophages because they also contained sphingosine 1-phosphate (S1P), a ceramide metabolite that activates its receptor in macrophages. They also showed increased C16:0 ceramide in the blood of mouse and humans with NASH. These data provide a mechanistic association between lipotoxic ER stress and disease pathogenesis via EVs and suggest that C16:0 ceramide and S1P content in EVs might be used as biomarkers in NASH patients. In the same line, Hirsova et al. have reported that, upon toxic lipid overload, hepatocytes can initiate a C/EBP homologous protein (CHOP)/death receptor 5 (DR5)/caspase-8/caspase-3 signaling cascade resulting in the activation of Rho-associated protein kinase 1 (ROCK1) and the release of EVs expressing TRAIL on their surface (33). Hence, TRAIL-bearing EVs were able to activate mouse bone marrow–derived macrophages toward an inflammatory phenotype (M1) via nuclear factor κB (NF-κB) signaling. They also showed that the release of Hep-EVs and, therefore, macrophage activation, was decreased by inactivating DR5 signaling pathway or using ROCK1 inhibitors. Likewise, they found that ROCK1 inhibition in mice with NASH led to a reduction of circulating EV levels associated with less liver damage such as inflammation and fibrosis. Moreover, Guo et al. (52) conducted a study that provides insights regarding the mechanism by which lipotoxic Hep-EVs may regulate peripheral blood monocyte adhesion to LSECs and hepatic recruitment and retention during NASH. They found that integrin β1 (ITGβ1), a cell adhesion molecule highly expressed in hepatocytes, plays a role in the progression of NASH. Lipotoxic insult in hepatocytes activates ITGβ1 and facilitates its endocytic trafficking and release of EVs, thereby promoting monocyte adhesion to LSECs, an essential step in hepatic inflammation. They also showed that blocking ITGβ1 in mice fed a FFC diet ameliorates liver inflammation, injury, and fibrosis. Hence, these authors propose that reducing the ability of LSECs to recruit harmful proinflammatory monocytes through ITGβ1 inhibition may serve as an anti-inflammatory therapeutic strategy to combat NASH. On the other hand, Cannito et al. demonstrated that EVs released by fat-laden hepatocytes undergoing lipotoxicity can directly activate the multiprotein platform complex nucleotide-binding oligomerization domain-like receptor protein 3 (NLRP3) inflammasome in both hepatocytes and macrophages, resulting in caspase 1 activation and pro–interleukin-1β and pro–interleukin-18 production, ultimately leading to a significant release of IL-1β (53). Since the release of EVs from lipotoxic cells and the subsequent activation of the NLRP3 inflammasome have been suggested to contribute to NAFLD progression, these data point to a novel rational link between lipotoxicity and inflammatory responses.

It is also noteworthy to mention a recent study conducted by Liu et al. showing that lipotoxic hepatocytes release exosomes enriched in miR-192-5p that activate proinflammatory macrophages and hepatic inflammation through the negative regulation of Rictor/Akt/FoxO1 signaling pathway (54). Furthermore, in patients with NAFLD, serum miR-192-5p levels positively correlated with hepatic inflammatory activity score and disease progression. Likewise, serum miR-192-5p levels and the number of M1 macrophages, as well as the expression levels of hepatic proinflammatory mediators, were correlated with disease progression in high-fat, high-cholesterol diet (HFHCD)–induced NAFLD in rats. Thus, they suggested serum exosomal miR-192-5p as a potential noninvasive biomarker and therapeutic target for NASH.

#### Hep-EVs Involved in Angiogenesis

Angiogenesis is a pathological feature of NASH and plays a central role in NAFLD progression. However, angiogenesis-inducing molecular and signaling mechanisms, as well as the potential link between lipotoxicity and angiogenesis, remain incompletely understood.

A study reported by Povero et al. (55) provided evidences that hepatocytes overloaded with saturated lipotoxic FFAs secrete proangiogenic signals. They identified Hep-EVs laden with vanin-1 (VNN1), an epithelial ectoenzyme with recognized cell migration and adherence properties, which induced endothelial cell (EC) migration and vascular tube formation, two processes required for angiogenesis. Of relevance, EVs derived from VNN1-deficient HepG2 cells failed to induce significant EC migration and tube formation. Likewise, administration of siRNA targeting VNN1 to mice fed with a methionine- and choline-deficient diet protected against NASH-induced pathological angiogenesis in the liver. Altogether, these findings uncovered a mechanism linking hepatocyte lipotoxicity to angiogenesis and identified a potential therapeutic target for developing novel antiangiogenic strategies for the treatment of NASH, as well as a circulating biomarker of liver damage.

#### Hep-EVs Involved in Fibrosis

Hepatic stellate cells play a crucial role during liver fibrosis in advanced NAFLD (56). When hepatic steatosis develops, HSCs are activated and express several fibrosis markers such as transforming growth factor β, tissue inhibitor of metalloproteinases 1 and 2 (TIMP-1 and TIMP-2), and matrix metalloproteinase-2 (57). However, the trigger for HSC activation in NAFLD is still under investigation. The following mentioned studies have suggested that EVs may have important roles in the crosstalk between hepatocytes and HSCs in the progression from simple steatosis to NASH, identifying potential molecular targets for antifibrotic therapeutic interventions.

As shown by a marked up-regulation of profibrogenic genes including collagen-I, α-smooth muscle actin, and TIMP2, as well as proliferation, chemotaxis, and wound healing responses, the study of Povero et al. (55) demonstrated that EVs released from lipid-laden hepatocytes are internalized into HSCs, inducing a phenotypic switch from quiescent to activated HSCs (required for development of liver fibrosis). These changes were associated with the EVs cargo miR-128-3p, which regulates several proteins involved in liver fibrosis and HSC activation, as well as peroxisome proliferator-activated receptor γ (PPAR-γ) that has been proposed as mediator in the maintenance of a quiescent HSCs phenotype in normal liver (58). Interestingly, exposure of HSCs to miR-128-3p–depleted EVs resulted in downregulation of profibrogenic markers and upregulation of PPAR-γ. Likewise, miR-128-3p–depleted EVs attenuated HSC proliferation and migration. Along these lines, Lee and colleagues have shown enhanced exosomes production and altered exosomal miRNA profile in palmitic acid (PA)–treated hepatocytes that increased the expression levels of fibrotic genes in HSCs. A step further, they confirmed that in exosomes from PA-treated cells, the expression of miRNA-122, one of the most abundant miRNAs in the liver (59, 60), was increased together with miRNA-192 also associated with NASH progression and fibrosis (61, 62). In this study, it was found that direct transfection of miRNA-192 into HSCs increased the expression of fibrosis markers. On the other hand, they found that the expressions of miRNA-122 and miRNA-192 were increased in circulating exosomes from patients with advanced NAFLD compared to those at early stages of the disease (63). Therefore, it was suggested that miRNA profiling in circulating exosomes may serve as a biomarker for the diagnosis of advanced NAFLD or NASH.

In summary, these relevant studies point to the participation of Hep-EVs in the modulation of responses of nonparenchymal cells of the liver including LSECs, HSCs, and KCs as a multiple-hit mechanism resulting in accelerated NASH progression.

### Hep-EVs and NAFLD-Associated Complications

The harmful effect of NAFLD is not only limited to damage of the well-known liver functions in metabolism and detoxification processes, among others, but also provides an independent risk for development of atherosclerosis and other related CVDs, which represent the main cause of death in these subjects (64). Although clinical evidences have linked NAFLD and CVD, the underlying molecular mechanisms need to be deciphered. The potential role of Hep-EVs in endothelial inflammation and atherogenesis in the context of NAFLD has been achieved by Jiang et al. (65). They identified miR-1 as mediator of the proinflammatory effect of EVs via downregulation of Kruppel-like factor 4 (KLF4), a transcriptional regulator of vascular homeostasis, and activation of the NF-κB pathway in ECs. Moreover, inhibition of miR-1 with a specific antagomiR-1 in an animal model of atherosclerosis accompanied by fatty liver (ApoE^−/−^ mice fed an high-fat diet) strongly suppressed vascular smooth muscle cells growth, stabilized plaques, and reduced endothelial inflammation leading to a marked amelioration of atherosclerotic plaque formation. This study provides convincing evidence implicating Hep-EVs in the distant communication between the liver and vasculature in NAFLD, and also unravels a molecular mechanism underlying the development of cardiometabolic disease.

In a different line and as previously mentioned, hepatic steatosis through aberrant accumulation of TGs in hepatocytes is the first hit during NAFLD development. Communication among metabolic tissues such as liver and adipose tissue regulates TG distribution in the body, which is critical for maintaining whole-body metabolic homeostasis (66). A recent study suggests that, in the context of lipid overload, the liver orchestrates the cross-talk with adipose tissue via specific EVs-containing miRNAs (67). They propose an interorgan mechanism whereby the liver in response to lipid overload sends an early signal to adipose tissue to modulate metabolic adaptations in order to counteract the excess of lipid deposition and also drives TG redistribution to maintain systemic homeostasis. At the molecular level, this study involves geranylgeranyl diphosphate synthase (GGPPS), an enzyme of the mevalonate pathway, which plays an important role in regulating glucose homeostasis and insulin sensitivity, in the secretion of Hep-EVs containing miRNAs. It was demonstrated that *Ggpps* expression is induced in hepatocytes by acute and chronic HFD consumption allowing geranylgeranylation of the Rab-GTPAse Rab27A, which, in turn, increases EV secretion. Among EVs-derived miRNAs, let-7e-5p enhances adipocyte lipid deposition by increasing lipogenesis and inhibiting lipid oxidation through alet-7e-5p-Pgc1α axis. Furthermore, this phenomenon is inhibited in liver-specific *Ggpps* knockout mice due to reduced Hep-EV secretion. Thus, this seminal study highlights a Hep-EVs–mediated liver–adipose tissue signaling axis that may be necessary for the metabolic adaptations of adipocytes to face lipid overload in order to maintain systemic homoeostasis during NAFLD.

## Circulating EVs as Biomarkers of NAFLD Diagnosis

As stated above, liver biopsy remains the gold standard procedure for diagnosis, staging, and monitoring of NAFLD. However, it is expensive, highly invasive, and inaccurate due to error sampling and carries some morbidity and a rare mortality risk; therefore, it is currently unsuitable for routine use in individuals at risk of NAFLD (68, 69). The paucity of systematic screening for NAFLD has led to massive underdiagnosis in patients progressing to advance NASH or cirrhosis, more severe and irreversible stages of the disease.

Currently, several noninvasive methods are being used in clinical practice to assess NAFLD in order to mitigate the need for liver biopsy. These include imaging techniques such as magnetic resonance (MR)– and ultrasound-based elastography and analysis of serum hepatic enzymes as surrogate markers of liver inflammation and synthetic function. Nonetheless, these techniques lack sensitivity and specificity enough for detection of the early stages and do not always correlate with the severity or extent of hepatocellular injury and/or inflammation (70, 71). Ideal candidate markers should reflect not only the presence of NAFLD, but also its severity, which is vital for early diagnosis and grading progression (45). This diagnostic utility may be further projected to the treatment of NAFLD at early stages in order to decrease the incidence of NASH and cirrhosis.

In this context, circulating EVs may represent an optimal noninvasive blood-based biomarker or, so-called liquid biopsy, for NAFLD diagnosis (35). Table 1 summarizes potential EVs-based biomarkers for NAFLD. Several protein-based EV biomarkers have been introduced for NAFLD liver damage (72), NASH (33, 50), or HCC (78, 80–82), although to date most studies have focused on characterizing EVs-associated nucleic acids, especially miRNAs and particularly in HCC (85–88). Apart from their use in liver malignancies, EVs-associated miRNAs may also serve as biomarkers in nonmalignant liver diseases such as NASH-induced fibrosis (84). Moreover, because of specific EVs-target cell–tissue interactions, lipid-based EV biomarkers might be important in NAFLD diagnosis as well, even though <3% of circulating lipids are transported in EVs (89). The only data available in this area is shown in the aforementioned study conducted by Kakazu et al. (51) showing increased C16:0 ceramide-enriched EVs in mice and humans with NASH. Nonetheless, circulating EVs are heterogeneous and do not exclusively reflect the specific contribution of the liver. Extrahepatic EVs may, in fact, mask liver-derived EVs, which ultimately, as we have reviewed, have a relevant role in NAFLD pathogenesis. Therefore, the identification of liver-specific markers in EVs might facilitate the detection of low-abundance cargos usually undetected, thereby providing direct information on disease progression, recovery, and treatment responses. In this regard, EV enrichment based on liver-specific markers followed by cargo analysis could represent a good strategy for biomarker discovery in NAFLD.

**Table 1:** Extracellular vesicle biomarkers in nonalcoholic fatty liver disease (NAFLD).

	**NAFLD stage**	**Vesicle source**	**Species**	**Sample**	**Biomarker**	**References**
Protein-based biomarkers	Liver damage	ND	Human	Serum, plasma	↑ sPTPRG	(72)
	NASH	ND	Mice	Plasma	↑ ASGPR1	(73)
		ND	Mice	Plasma	↑ CXCL10	(50)
		ND	Mice	Serum	↑ CYP2E1	(33, 50)
		Hepatocytes	Mice	Plasma	↑ VNN-1	(55)
		Hepatocytes	Mice	Plasma	↑ ASGR1, CYP2E1	(74)
		Macrophages	Mice	Plasma	↑ Gal3	(74)
		Neutrophils	Mice	Plasma	↑ Ly6G	(74)
		Leukocytes	Human	Serum, plasma	↑ CD14, iNKT	(75, 76)
	Cirrhosis	Hepatocytes	Human	Plasma	↑ CK18	(77)
	HCC	Hepatocytes	Human	Plasma	↑ Hep Par 1	(78, 79)
		ND	Human	Serum	↑ EpCAM, CD133, ASGR1	(80)
		ND	Human	Plasma	↑ANXA2	(81)
		ND	Human	Serum	↑ PIGR	(82)
		ND	Human	Serum	↑ LG3BP	(82)
Nucleic acid–based biomarkers	NASH	Hepatocytes	Rat, human	Serum	↑ miR-192-5p	(54)
		Hepatocytes	Mice	Plasma	↑ miR-122, miR192	(73)
		Hepatocytes	Human	Serum	↑ miR-122, miR192	(63)
		Hepatocytes	Mice, human	Plasma	↑ MitoDNA	(83)
	Fibrosis	ND	Mice	Serum	↓ miR-214 (↓Twist1 ↑CCN2)	(84)
	HCC	ND	Human	Serum	↑ miR-21	(85)
		ND	Human	Serum	↓ miR-718	(86)
		ND	Human	Serum	↑ miR-18a, miR-221, miR-222, miR-224	(87)
		ND	Human	Serum	↓ miR-101, miR-106b, miR-122, miR-195	(87)
		ND	Human	Serum	↑ miR-939, miR-595, and miR-519d	(88)
Lipid-based biomarkers	NASH	ND	Mice, human	Plasma	↑ Ceramide and S1P	(51)

*ANXA2, annexin A2; ASGR1, asialoglycoprotein receptor 1; CCN2, connective tissue growth factor; CD133, cluster of differentiation 133; CD14, cluster of differentiation 14; CK18, cytokeratin 18; CXCL10, C-X-C motif chemokine ligand 10; CYP2E1, cytochrome P450 family 2 subfamily E member 1; EpCAM, epithelial cell adhesion molecule; Gal3, galectin 3; HCC, hepatocellular carcinoma; Hep Par 1, hepatocyte paraffin 1; iNKT, invariant natural killer T; LG3BP, galectin-3-binding protein; Ly6G, lymphocyte antigen 6 complex locus G; miR, mature form of the miRNA; NAFLD, nonalcoholic fatty liver disease; NASH, nonalcoholic steatohepatitis ND, not defined; PIGR, polymeric immunoglobulin receptor; S1P, sphingosine 1 phosphate; sPTPRG, soluble protein tyrosine phosphatase receptor gamma; VNN1, vanin-1*.

### Circulating Liver-Derived EVs

One of the first studies to overcome this challenge was conducted by Povero et al. in experimental murine models of diet-induced NAFLD and early- and advanced-NASH. These authors observed that levels of circulating EVs increased over time during NASH progression and strongly correlated with several histological features such as cell death, angiogenesis, and fibrosis (73). Consistent with previous findings of other groups (90, 91), in a subsequent analysis Povero et al. revealed that circulating EVs isolated from mice with NAFLD were enriched in miR-122 and miR-192, two miRNAs abundantly expressed in hepatocytes. Based on these results, they proposed that hepatocytes were likely the main source of circulating EVs, concluding that Hep-EVs increased in mice with NAFLD (73). Extracellular vesicle–associated miR-122 and miR-192 were later validated by Lee et al. (63) as a biomarker of NASH progression in sera from NAFLD patients. Nevertheless, Hep-EVs as noted earlier were also enriched in the enzyme VNN-1, which promoted angiogenesis and induced liver damage in NASH, hence representing another potential biomarker (55). Furthermore, in the mentioned study of Liu et al., an increase in Hep-EVs and EV-associated miR-192-5p in serum of HFHCD-fed rat NAFLD model and NASH patients was also observed, therefore proposing miR-192-5p as other biomarker (54). In a previous study, we found that hepatocyte-derived circulating EVs containing mitochondrial DNA were also increased in plasma from both mice and patients with NASH, and importantly, this work implicated EVs in macrophage activation via Toll-like receptor 9 (TLR9) (83).

A distinct approach to identify liver-derived EVs was achieved by Brodsky et al. (79). They isolated circulating EVs enriched in proteins from liver origin in patients with HCC using flow cytometry and immunolabeling against the protein hepatocyte paraffin 1 (Hep Par 1), an antibody to carbamoyl phosphate synthetase 1 used as tissue marker of HCC. These authors found increased levels of circulating liver-derived EVs in patients with HCC that correlated with the size of the liver tumors. Endothelium-derived EVs were also evaluated, and the same correlation was found (79). In this line, more recently Li et al. (74) identified Hep-EVs using nanoscale flow cytometry detecting hepatocyte selective surface markers on EVs such as asialoglycoprotein receptor 1 (ASGR1) and cytochrome P450 family 2 subfamily E member 1 (CYP2E1). By using this technology, they found increased Hep-EVs in both male and female mice at an early NAFLD stage (12 and 10 weeks of FFC diet feeding) before histologically apparent inflammation and remained elevated over time (24, 36, and 48 weeks). Macrophage- and neutrophil-derived EVs were also analyzed due to the important role of the immune signature in NASH. Macrophage- and neutrophil-derived EVs were significantly elevated at 24 weeks of dietary feeding concomitant with histologic inflammation in the liver. Furthermore, hepatocyte-, macrophage-, and neutrophil-derived EVs strongly correlated with the histologic assessment of NASH and noninvasive MR-based biomarkers of NASH. They also quantified platelet-derived EVs demonstrating sexual dimorphism because they were elevated in male mice at 12, 24, and 48 weeks of dietary feeding, whereas in female mice elevations were found at 24 weeks (74). This work constitutes the first descriptive report of the kinetic changes in hepatocyte-, macrophage-, neutrophil-, and platelet-derived EVs in a mouse model of NASH.

### Circulating Immune Cells–Derived EVs

Regarding the immune system in NAFLD progression, it is important to highlight the pioneering study of EVs-based NAFLD diagnosis in humans published by Kornek et al. (75). They suggested for the first time the existence of a correlation between the circulating abundance of specific leukocyte-derived EVs and disease severity, as determined by liver transaminase levels, biopsy grade, and NAFLD activity score. To date, these findings still represent the most compelling study with clinical samples for NAFLD diagnosis and progression based on EVs. Consistently, this study was recently confirmed by Welsh et al. (76), who also reported leukocyte-derived EVs as a marker for liver fibrosis severity in NAFLD. It is noteworthy to mention that Kornek et al. (75) also observed that patients with chronic hepatitis C could be differentiated from patients with NASH using immune cells–derived EVs. This was further supported by another study in which transcriptomic analysis revealed that serum EVs-derived miRNAs were regulated either positively or negatively with the histological features of the disease such as inflammation and fibrosis, therefore differentiating multiple etiologies of liver disease, as well as disease from healthy controls (92). In another study, both circulating Hep-EVs and immune cell–derived EVs were analyzed (77). Extracellular vesicles from hepatocytes or myeloid origin were found increased in patients with cirrhosis compared with healthy individuals. In patients with cirrhosis, plasma Hep-EVs contained high levels of cytokeratin-18 compared with healthy individuals. Moreover, the severity of cirrhosis correlated with the levels of leukoendothelial EVs and Hep-EVs (77).

Taken together, all these studies have established a solid background for EV biomarker discovery in NAFLD diagnosis. However, because of the current notable limitations, there is still a long way to go before EVs-related assays will have translational utilities. Aside from disease and tissue specificity, the lack of generally accepted standardization of the methods for EV isolation and guidelines related to sample collection and handling can interfere with downstream analysis, resulting in high variability that complicates the reproducibility and validation of EVs as biomarkers (35, 45).

## Concluding Remarks

In this review, we have summarized some of the most recent and original studies investigating the key role of EVs released by stressed hepatocytes (Hep-EVs) by targeting nonparenchymal cells such as HSCs, LSECs, and macrophages. This interactome links lipotoxicity with inflammation and angiogenesis, relevant events in the progression of NAFLD to NASH stage. In addition, we compiled several studies on the significant interest of Hep-EVs released into the systemic circulation as potential biomarkers for NAFLD diagnosis and progression. Future studies to examine additional molecular mechanisms involved in EVs biogenesis, release, and dysregulation of target cells, as well as the identification of cargos with potential value as biomarkers for noninvasive diagnosis and monitoring of disease progression, are highly awaited.

## Author Contributions

IG-M, RA, PR, and AV contributed to the preparation and writing of this review article. All authors contributed to the article and approved the submitted version.

## Conflict of Interest

The authors declare that the research was conducted in the absence of any commercial or financial relationships that could be construed as a potential conflict of interest.

## Abbreviations

ASGR1: asialoglycoprotein receptor 1
CHOP: C/EBP homologous protein
CVD: cardiovascular disease
CXCL10: C-X-C motif chemokine ligand 10
CYP2E1: cytochrome P450 family 2 subfamily E member 1
DAMPs: Damage-associated molecular patterns
DR5: death receptor 5
EC: endothelial cell
ER: endoplasmic reticulum
EVs: extracellular vesicles
FFAs: free fatty acids
FFC diet: fat-fructose- and cholesterol-enriched diet
GGPPS: geranylgeranyl diphosphate synthase
HCC: hepatocellular carcinoma
Hep-EVs: hepatocyte-derived EVs
Hep Par 1: hepatocyte paraffin 1
HFHCD: high-fat, high-cholesterol diet
HSCs: hepatic stellate cells
IL: interleukin
IRE1α: inositol requiring enzyme 1α
ITGβ1: integrin β1
ILV: intraluminal vesicle
KCs: Kupffer cells
KLF4: Kruppel-like factor 4
LSECs: liver sinusoidal endothelial cells
miR: mature form of the miRNA
MISEV: Minimal Information for Studies of Extracellular Vesicles
mitoDNA: mitochondrial DNA
MLK3: mixed lineage kinase 3
MR: magnetic resonance
MVBs: multivesicular bodies
NAFL: nonalcoholic fatty liver
NAFLD: nonalcoholic fatty liver disease
NASH: nonalcoholic steatohepatitis
NLRP3: nucleotide-binding oligomerization domain-like receptor protein 3
PA: palmitic acid
PPAR-γ: peroxisome proliferator-activated receptor gamma
ROCK1: rho-associated protein kinase 1
S1P: sphingosine 1-phosphate
T2DM: type-2 diabetes mellitus
TG: triglycerides
TIMP: tissue inhibitor of metalloproteinases
TLR9: Toll-like receptor 9
TRAIL: tumor necrosis factor-related apoptosis-inducing ligand
VNN1: vanin-1.
